# Teasing apart “the tangled web” of influence of policy dialogues: lessons from a case study of dialogues about healthcare reform options for Canada

**DOI:** 10.1186/s13012-017-0627-3

**Published:** 2017-07-28

**Authors:** Gillian Mulvale, Samantha A. McRae, Sandra Milicic

**Affiliations:** 1 DeGroote School of Business, McMaster University, Ron Joyce Centre Room 421, 4350 South Service Road, Burlington, ON L7L 5R8 Canada; 2 DeGroote School of Business, McMaster University, 1280 Main St W, Hamilton, ON L8S 4L8 Canada; 3 Propel Centre for Population Health Impact, University of Waterloo, 200 University Avenue, Waterloo, ON N2L 3G1 Canada

**Keywords:** Policy dialogue, Knowledge exchange, Capacity development, Influence on policy-making, Policy stages, Conceptual framework, Policy environment

## Abstract

**Background:**

The knowledge exchange literature suggests that policy dialogues are intended to enhance short-, medium- and long-term capacities of individuals, organizations and health systems to use evidence to inform policy-making. Key features of effective dialogues have been suggested, but the linkages between these features and the realization of improved capacities for evidence-informed policy-making among dialogue attendees and the subsequent influence on policy-making activities are not well understood.

**Methods:**

We conducted a qualitative case study of a series of four policy dialogues that were convened in Canada among national, provincial and regional stakeholders on topics pertaining to healthcare financing and funding in 2011. Data sources included videos of participant perspectives captured during or immediately following each event and follow-up key informant interviews among dialogue participants held 4 years later in 2015. Three conceptual frameworks pertaining to (i) policy dialogues and capacities for evidence use, (ii) factors shaping policy-making across the policy cycle and (iii) factors shaping implementation of evidence guided the thematic analysis. We then synthesized the findings across the three frameworks.

**Results:**

The results suggest the potential benefits of policy dialogues described in the literature were developed among the participants at these dialogues. Informants elaborated on how dialogue features influenced their capacities to use evidence, the ideas, interests and institutions during the agenda-setting and policy formulation stages of policy-making and how implementation was affected by characteristics of policy options, individuals, organizations, the external environment and processes.

**Conclusions:**

We present a conceptual framework that furthers our understanding of the potential influence of policy dialogues on the content and mechanisms of policy development and illustrate pathways of influence on various stages of the policy cycle from agenda setting through formulation and implementation. The framework highlights important factors for consideration in designing and evaluating policy dialogues and in supporting post-dialogue knowledge exchange efforts.

## Background

Policy dialogues are a knowledge transfer and exchange approach [1–3] that has been used at the national level in Canada to shine a spotlight on areas for potential health policy reform [4–8] in a context where provincial and territorial governments are responsible for policies pertaining to healthcare delivery. Policy dialogues are group processes that provide opportunities for interactive knowledge-sharing that bring research evidence together with the views, experiences and tacit knowledge of those involved in or affected by future decisions about high-priority issues [9]. By promoting interactions between researchers and policy-makers (elected or appointed official who set the agenda and propose, develop and evaluate policies), policy dialogues can help in identifying and interpreting available evidence and finding areas of accord between research evidence and policy-makers’ beliefs, values, political goals and strategies [4, 10].

A key feature of effective policy dialogues is their deliberative nature. Deliberative approaches foster structured conversations that value listening as much as speaking, informed and reasoned argument to develop collaborative understanding of the values that may underlie opposing views, and weigh reasons for and against different policy options with the aim of building toward common action [11]. These approaches enable participants to explore diverse views, build trust, connect research to personal experience, and develop agency to take action within their own spheres [11–13]*.* With the right mix of participants, appropriate use of evidence, and a conducive dialogue setting, Boyko and colleagues suggest dialogue participants may enhance their capacities (knowledge, abilities, skills and actions to meet goals) [8] to use evidence in policy-making in the short term. These enhanced individual capacities contribute to enhanced organizational and system capacities over the medium and longer terms respectively [8].

Better understanding is needed of how the capacities gained through attending policy dialogues can contribute to evidence use in policy-making processes. These processes refer to how government and organizational policy decisions are made, where policy refers to statements of collective decisions, actions, processes or goals. The policy literature describes a policy cycle [14] that includes defining problems [15, 16] and agenda setting [17], policy formulation, decision-making, implementation and evaluation [18] which may or may not occur sequentially. Each stage is subject to complex interacting elements that can influence policy progression and can similarly influence the effect of any given policy dialogue [8]. For example, the 3-I framework (ideas, interests and institutions) recognizes that research evidence is only one consideration when it comes to *ideas* in policy-making; values, goals and perceptions of stakeholders (individuals who may be representative of governments, interest groups, non-governmental organizations) [16] their relative strength, relationships and positions (*interests*) on different policy issues, and *institutions* such as laws, regulations and tradition are also influential [19]. For example, Canada’s complex institutional landscape means the federal role has traditionally been set to guide principles and contribute funding toward provincially run publicly financed health insurance programs for hospital and physician services and deliver care to specific populations (e.g. First Nations, armed forces, federal penitentiary) [20, 21]. Any policy dialogue intended to influence policy-making must reflect this context.

The implementation science literature similarly describes the implementation of research into practice as a “complex and messy task” [22] that depends on many factors including evidence, context and factors that facilitate the change process [23]. While a recent systematic review identified 61 theories and frameworks (models) to enhance the spread of evidence-based interventions, only two models focused on implementation and policy level considerations [24]. The proponents of one of these models [10] point to the importance of better understanding how ideas are diffused across policy-making. The other model, the Consolidated Framework for Implementation Research (CFIR) [25], offers a consolidation of key constructs from published implementation theories as a “pragmatic structure for approaching complex, interacting, multi-level and transient states of constructs” [26]. It can be combined with the 3-I and policy cycle frameworks to better understand how policy dialogues can influence evidence use in policy-making.

This study used data from follow-up key informant interviews with delegates who attended four policy dialogue events hosted in 2011 by the Canadian Foundation for Healthcare Improvement (CFHI), as well as video commentary captured at the events. The goal was to better understand the extent to which the intended effects of policy dialogues on capacity development described in the literature were borne out in practice [27] and to learn from participants whether, and if so how, enhanced capacities influenced their subsequent activities in the policy realm across the various stages of the policy cycle. The findings contribute to the literature about the capacities that can be gained by participating in a policy dialogue and elaborate the mechanisms by which those capacities may influence policy processes and implementation of reform options. Better understanding of these mechanisms offers the opportunity to enhance the design and use of policy dialogues as knowledge exchange vehicles to foster use of evidence in policy-making.

## Methods

We conducted a case study [28] to explore the role of policy dialogues in knowledge uptake and use by policy decision-makers and stakeholders based on the perspectives of participants at such events. The case study approach is particularly well suited to investigate “a contemporary phenomenon within it’s real-life context” [28] and can help to draw lessons to improve practices in other cases [29]. It typically involves the use of a priori theory or a conceptual framework to improve rigour in data-gathering and analysis [30, 31] and allows theory to evolve based on emerging findings [31].

The case is defined to include the influence (if any) on subsequent policy processes of four policy dialogue events within the Healthcare Financing and Transformation (HFiT) dialogue series held by CFHI in 2011: three half-day stakeholder dialogues on healthcare financing and payment with approximately 20 to 25 participants each and the International Health Economics Association (iHEA) pre-conference symposium with 92 participants and an international expert panel [32]. These policy dialogues were selected because (i) they followed similar formats that met the effectiveness criteria spelled out in the literature [8]; (ii) they included national, provincial and regional policy-makers and stakeholders in Canada (see Table 1); (iii) sufficient time had passed that research informants could comment on policy influence over the short- and medium-terms, while still being able to recall the events; and (iv) they focused on a high-priority issue (healthcare financial sustainability) that was expected to influence policy-making because of anticipated federal-provincial Health Accord negotiations.

**Table 1:** HFiT dialogue participants classified by policy role

Event:	Stakeholder dialogue 1	Stakeholder dialogue 2	Stakeholder dialogue 3	iHEA symposium
Topic	Pharmaceutical pricing and health technology assessment	Hospital funding and physician remuneration	Healthcare financing and social insurance	Policy options to improve healthcare sustainability
Participants:				
Policy-maker—federal	3	2	1	2
Policy-maker —provincial^a^	1	6	4	6
Other stakeholders—national^b^	15	8	13	24
Other stakeholders—provincial	0	7	2	8
Researchers	3	2	4	21
International	0	0	0	31
Total	22	25	24	92

^a^Ministries may include health, finance, pharmaceutical policy

^b^Stakeholders may include representatives of health professional organizations, patient organizations, hospital associations, academic health science, disease-related organizations, accreditation and other national health policy organizations

Three conceptual frameworks were used as guides to the research. As discussed above, the Boyko model [8] relates features of policy dialogues (setting, mix of participants, use of evidence) to capacities of individuals, organizations and systems to engage in evidence-informed policy-making at each level over the short, medium and long terms respectively. The 3-I framework describes factors that influence policy development across the various stages of the policy cycle. The CFIR model describes characteristics of the intervention and implementation processes and contextual factors at multiple levels (individual, organizational, external policy and systems) that influence implementation of evidence-based options.

**Fig. 1:**
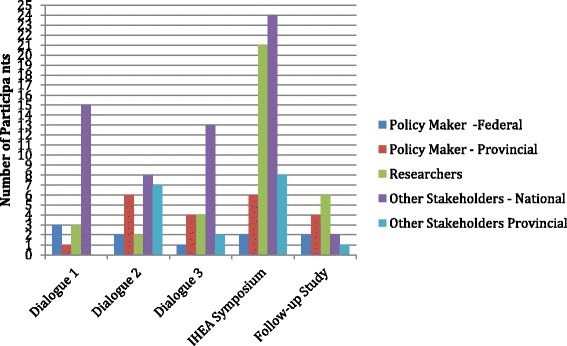
HFiT policy dialogue participants by dialogue and participant type

Data sources in this study included: (i) eight video recordings of commentary from 27 dialogue participants who volunteered to share their perspectives immediately after the initial events, (ii) 10 follow-up key informant telephone interviews with policy-makers and other stakeholders and (iii) a follow-up telephone focus group interview involving five researchers who had presented policy options at the dialogues. Most research attendees were presenting policy options for other participants to respond to. To capture diverse government, stakeholder and researcher perspectives, the lists of attendees of the dialogues were purposively sampled. As can be seen from Fig. 1, the numbers of participants by type varied by dialogue topic and event format. In the first three dialogues, participation was by invitation. The first dialogue focused on pharmaceutical pricing and health technology assessment at the national level and so had more national stakeholders compared to other dialogues. The second dialogue focused on hospital funding and physician payment and so included relatively greater provincial participation. The third dialogue on healthcare financing had relatively high national representation because of the joint federal-provincial role in healthcare financing in Canada. The iHEA symposium was a larger international event open to attendees of a research conference and so drew a larger proportion of researchers and national stakeholders.

Interviews were held between May and June 2015. Semi-structured and focus group interview guides asked informants to reflect on their experiences at the dialogues, the helpfulness of attending the dialogue, and any influence of the dialogues on subsequent policy processes. The interviews averaged 45 min in length, and the focus group lasted 60 min. Data were professionally transcribed.

The thematic coding of the interview, focus group and video data proceeded through two stages. Research team members initially reviewed all the data to obtain an overall impression of the findings. A subset of transcripts was then thoroughly reviewed by two researchers to reveal common themes. There was a very high level of agreement across coders (85%) based on an initial sample of 10% of coded content. The resulting codebook was used to analyse the remaining transcripts. We focus in this paper on comments about the influence of the dialogue on capacities, actions taken and influence on subsequent policy-making rather than on participants’ views of the policy options themselves.^1^ We included any subthemes within the Boyko model (e.g. enhanced mutual understanding as an individual capacity) that were mentioned by a minimum of two informants. During this stage of coding, we identified emergent themes that pertained to how those capacities were shaped by the dialogue and any subsequent influence on policy-making (e.g. seeding new *ideas*) within the 3-I framework during the agenda setting and formulation stages of the policy cycle as discussed in the Boyko model. In the second stage, team members recoded the data using the categories of the CFIR model to reflect informant statements about contextual factors that could shape implementation of the options discussed at the dialogues [26].

In the final stage of analysis, we synthesized the various themes to develop a conceptual framework and set of illustrative figures that propose pathways by which policy dialogues can influence capacity for evidence-informed policy-making at different stages of the policy cycle. Informants had an opportunity to review relevant portions of the thematic analysis and quotes attributed to them to ensure their accuracy. Ethics approval was granted from the McMaster University Research Ethics Board.

## Results

We first discuss the thematic analysis across using each of the conceptual frameworks. Tables 2, 3, 4 and 5 provide illustrative quotes for each theme and subtheme, which are italicized in the text that follows. We begin with dialogue features (Table 2) and influence on capacity development (Table 3) consistent with the themes and subthemes of the Boyko framework [8]. We then discuss emergent subthemes about how the capacities that were developed influenced subsequent activities in the policy domain using the 3-I [19] (Table 4). We next discuss contextual factors that influence implementation using the CFIR framework [26] (Table 5). We then present a thematic synthesis that integrates themes across the three models, a conceptual framework, and propose potential pathways of influence of policy dialogues on different stages of policy-making. We indicate the informant type abbreviation (see details in the “Abbreviation” section) and number in square brackets following each quote. For example, R02 refers to the second researcher informant.

**Table 2:** Statements about dialogue features

Environment
“What I liked particularly about today is that it wasn’t merely the transfer uh or the communication of a set of conclusions it was actually much more generative then that. [PPM1]
“I think that these open forums that focus on specific policy issues and go through challenges, solutions, and opportunities. I think that sort of thing should happen more often --” [PPM2]
“Well what I think was really unique about today’s symposium was the opportunity to consider several options at the same time with the same group of people”. [R6]
“I think the single most thing- most surprising thing I found about today was just how long and how engaged people stayed throughout the entire day. These sorts of symposiums can very quickly become exercises in attrition but we started with pretty much the same group of people we finished with.” [PPM1]
Participants
“I think it’s valuable to have those different players in the room like the medical association, presidents, or CEO’s along with the policy [advisors] because a lot of us are academics and focus more on solutions on how to do this stuff rather than this whole thing be a show and tell for a particular government on their new policy.” [PPM2]
“Having senior leaders from different organizations, whether it was a regional health authority or a deputy minister within government, you have individuals who truly can make big thing decisions about-- or influence the future organization management delivering financing of the respective health system. From that standpoint of that I found it very strong.” [NSO2]
“… you had some very different players that probably normally wouldn’t get together, who would be on opposite sides of negotiating tables, for example -- the membership, the event was great. But the participants, I think it was set up well to bring up all those different perspectives and [promote] natural discussion.” [PPM2]
Evidence use
“Yes and I think um that really is why today’s symposium was such a unique opportunity for to do that, to reach out around the world and hear about practice in other countries. Certainly all of [the] knowledge synthesis that [were] commissioned … look to the international evidence that is published in the literature, but by having people from other countries in the room … that can talk from their own experience within a country … goes beyond what you can capture in a published journal article to have that kind of experiential knowledge in the room as well.” [R6]
“And what I think I took away from today was both the value of the evidence but the value of having discussion around the evidence so that people start to come up with recommendations, findings, conclusions that go far behind what was included in any of the reports on their own.” [PPM1]

**Table 3:** Statements about strengthened capacities for evidence-informed policy-making from attending dialogues

individual capacities	
Mutual understanding	“I hope that we did bring enough of the policy wonk types, and the academics, and the health people together to walk away … and then after some connections with someone else, they get to a place they weren’t before. So that’s my opinion about any kind of policy workshop. It just gets people in a room and builds trust and cohesion, and it gets people focused on common issues.” [R7]
New ways of thinking	“The exchange allows here just an exchange of ideas. So hopefully put other than your own thoughts and our own views, based on where we worked or how we see things functioning. I would hope that it’s kind of broadened everyone’s mind about different ways to do things.” [NSO2]
Relationships	“I suspect that the communities have been strengthened, just by opening more effective lines of communication” [NSO2]
Empowerment	“We get the confidence about maybe the path we’re taking, or we change the path we’re taking based on what we learned. So in that sense, they’re useful.” [PPM3]
Organizational capacities	
Agenda setting	“… I probably would have mentioned something at some point where again, all of [province] is kind of implementing this stuff too right now … It might have been this sort of thing that these things rub off on other people there, help them advance their own agendas.” [PPM2]
Policy formulation	“But I think forums like this help us learn from each other find out what’s worked what isn’t working and not necessarily take something holis bolis from [one jurisdiction to the next] because they are different in structure and… or they might have some successes in [jurisdiction] to learn from and I think that is probably the best way to go forward.” [PPM3]
Preparation for policy windows	“As long as those windows are open, policy dialogues like this do play a role, because we get ideas from each other.” [PPM3]

**Table 4:** Influence on factors that affect policy-making

ideas	
Viewing the landscape (A)	“From my perspective in my role here, it’s really important for me to understand what kind of a landscape is out there of different views around issues. So that’s a key part of our policy assessment, of what ideas might fly and what ideas are likely not going to be supported.” [PSO2]
Seeding new ideas (A, F)	“The exchange allows here just an exchange of ideas. So hopefully put other than your own thoughts and our own views, based on where we worked or how we see things functioning. I would hope that it’s kind of broadened everyone’s mind about different ways to do things.” [NSO2]
Socializing ideas (A, F)	“I think policy dialogues like that, like these big large events are important because of how they socialize information, not because of how they influence decisions. When I was a policy-maker, the really important discussions for me often were very small groups, or Chatham House Rule type thing. Because then I could test out ideas, and I could see directly where stakeholders I had to get outside were going with this.” [PPM1]
Prioritizing options (A, F)	“As I listen to this, I understand the sophistication of the ideas, the relative sophistication of the ideas, I guess, that’s-- people are positioning around, and so on. It helps me kind of make my mind up about what I’m going to advocate about.” [PPM1]
Forming a tighter consensus (A, F)	“But what you see through a conference like that, is you see the broad socialization of idea and a chance to critique it. You see broad support for it, and perhaps as importantly, no one’s saying ‘No, don’t do that.’ Which makes it a lot safer for folks.” [PPM1]
Shaping options for discussion (F)	“I think by having a more detailed discussion like this you get down to some of the mechanics and then you realize these are some very different policies that [province] is doing… and I think you really need to get down to that more detailed level of the policy mechanics” [PB03SI]
Interests	
Creating a more cohesive policy community (A, F)	“I think events like that confirm that you weren’t alone in this, you weren’t crazy, certainly when you were-- you go and speak with people in the minister’s office and justify why you’re doing this or that. You could say, ‘Hey, the rest of Canada is thinking the same thing too.’ Since I was the only one from the … Ministry there, and I recall it was useful for me to be able to sort of reference that conversation to folks afterward, certainly” [PPM2] “It really, I think, led to just a more cohesive community, less confrontational, because more leaders from different sides of the equation knew each other. And it allowed for free-forum, free-flow discussion on a whole range of policy issues that, when you put people in rooms where they’re wearing their hats and their titles you don’t always have that kind of possibility.” [NSO2]
Face-to-face connection (A, F)	“So, it was more what I gained from those dialogues was more like a connection. I knew that there was an incentive somewhere and I could contact someone that would [?] me to the appropriate person.” [PPM4] “Honestly I think that the only role they bring is connecting people in a room.” [R7]
Will for change (A, F)	“Well for me as an observer and a participant there, the remarkable thing to me was that everybody knows what the problems are and it’s a lack of political will on the part of governments, on the part of physician representation organizations. Just everybody shares the blaming as far as the current system is concerned, and the notion of moving away from it.” [PSO2]
Institutions	
Centralized evidence-gathering (A, F)	“Perhaps something that actually transcends the political process as well, and not just collaboration among the provinces and territories, but really collaboration that sets a Canadian tone, a Canadian perspective - particularly on the evidence side. We kept on hearing the notion of the evidence perhaps collected at the national level, or pan-Canadian level, but perhaps interpreted at a provincial and territorial level” [PPM1].
Centralized benchmarks (A, F)	“…the policy makers said, ‘Well, why are you presenting option one, option two, option three? We need all these options together. We just need to see how we can put in place all the conditions and make sure that we can anticipate the different consequences of doing that and that and that, not taking them separately and analyzing the evidence on each aspect.’”[R16]
Common performance measures (F, E)	“There’s a few clinical indicators that we probably can say - it should be eight - but there’s relatively few. Good performance is what’s better than just about everyone else and what’s getting better over time. And so building on that, I take [event participant name]’s point. I’d say agree entirely now, and one of the most important things we could do is actually have comparable measures of performance, not just within a jurisdiction but across the jurisdictions in this country.” [PPM1]

*A* agenda-setting stage, *F* formulation stage, *E* evaluation stage of policy cycle

**Table 5:** Influence of policy dialogues on policy implementation

Intervention characteristics	
Evidence strength and quality	“So in my example, I think that’s certainly true where there has been no visible or no - at least what I’m aware of - significant discussion about the policy option. It may … be that the evidence … is so mixed that there isn’t a very strong case for taking it forward in a more practical or thinking about feasibility and implementation … it doesn’t mean there’s no impact [of the dialogue], it could mean that there was some impact.” [R1]
Relative advantage	“There’d be a couple things that’d be helpful at the discussion. One is, it did help me, as a whole, to understand the relative importance of different mechanisms, or the relative likelihood of payoff of the different mechanisms.” [PPM1]
Adaptability	“I don’t think we succeeded very well to try to imagine what would a social insurance model applied to healthcare look like in the Canadian context. We kind of keep defaulting back to countries where we observe that approach to financing healthcare and can’t seem to get to the point where we say, “Well, this is how you could construct something like that in the Canadian context whether you apply it to drugs, or mental health, or to the system as a whole.” So that one I think is really interesting. I don’t think the paper itself and by the dialogue … got us to where we could have gone.” [FPM1]
Design quality and packaging	“… any given dialogue helps contribute to developing a compelling package of evidence that might incrementally, over time, contribute to the policy-making process and in turn, the opening of a policy window for potential reform.” [PPM1].
Cost	“What is the opportunity cost? Which patient groups will lose out because the money for funding that has been taken from their account?” [R11]
Characteristics of individuals	
Knowledge and beliefs about the intervention	“It [the dialogue] probably just helped to nudge it along. I don’t think it’s hurt at all, the information was very useful - whether it was around strategy, tactics or system design - for certain individuals around the table. So I would say, if anything, it was again incremental, but generally that’s the way we make gains in our system. It is really incremental.” [NSO2]
Self-efficacy	“Well, you have to remember at the time, it was 2011, and at the time all of these things, what happens is people start feeling, at the end of these things, they all feel good. You know what I mean? Most of these meetings, they’re designed to make you feel good about yourself. And within the context of what’s going on at the time, that’s important because people can tell they get a little bit more confident.” [PPM3]
Outer setting	
Patient needs and resources	“The difficulty to get traction on change around, and excitement about, a national pharma-care plan. If you talk to Canadians about what their big problem is in terms of healthcare coverage, pharmaceuticals isn’t the issue that comes up. It has to do with other issues about access to a doctor, access to an emergency room” [R12]
Cosmopolitanism	“The dialogue creates such a face-to-face connection. I would say that I reached people in the various sectors when I needed to and it was easy to connect with them and ask them like, “Alberta, I remember you had this policy on long-term care. Can you refer me to someone that will be able to give me extra information on this and that? So it was more what I gained from those dialogues was more like a connection. I knew that … I could contact someone that would direct me to the appropriate person” [PPM4]
Peer pressure	“I think it was a topic area [hospital funding] that was right for action because Canada is sort of behind in most of the other countries in the developed world in changing how hospitals are funded. Pretty much every other country in the world has moved away from the block funding system that predominates in Canada, and so this is the topic area that’s right for change since we’re sort of, we haven’t evolved the way the rest of the world has.” [FPM2]
External policy and incentives	“The Federal Government has been less active, that’s a result of not engaging in the Accord. So that changing policy environment sort of had an effect of limiting the uptake of some of the-- but not eliminating, but it reduced the uptake of what was in the policy dialogues.” [FPM2]
Inner setting	
Structural characteristics	“…one of the things that I learned was the turnover in government, especially future government, is the half-life of those people is at least half of what it takes to make real change happen. So the turnover problem was huge. It was always huge, because new people have come who are in senior positions who I had to bring outside and educate, and just as soon as I’d finished doing that and they were outside, they were gone and somebody else came in and that was a constant problem.” [PPM3]
Culture	“… but the thing that’s preventing change, frankly it’s fear. It’s fear of doing anything different. Because all those things contain a risk. Every time you do something different, there’s a risk involved. And we have a very risk-averse structure here. Both inside health care, and at the political level - highly risk-averse. And the risk involves two different risks. One is the risk of adverse publicity. … And even good news in health care can turn to bad news overnight depending on how it’s played in the media. And I think governments have figured that out, and that’s why they’re backing off with health care. At every level, governments they’re trying to just distance themselves from anything having to do with health care as much as they can, so to stay away from hot arguments” [PPM3].
Implementation climate	
Tension for change	“So it’s not a shortage of knowledge. We don’t need to study what the problems are in health care, we actually have a really good idea of what the problems are, we know what most of the solutions would be, at least in a broad sense … [however] we won’t change health care dramatically until there’s a crisis because there’s no incentive to do so, and so health care at some point in time will fail catastrophically and what will come out of the other side will be worse for everybody, and I don’t think that’s going to be a surprise to anybody.” [PSO2]
Relative priority	“You could see when it was a policy option that was … embraced, that there was incentives to put them in place as opposed to other options where…they could see there were more limitations.” [PPM4].
Readiness for change	
Leadership engagement	“[the dialogue] … created this big buzz, and [province] got really attracted. The Minister … got really attracted. … It provides them also a momentum, which was really important for the Minister… they’ve been able to use this information … to move and to bring this to life.” [PPM4].
*Available resources*	“If it had happened like four or five years earlier where we had a lot of money to put on the table for that stuff, it’s probably feasible if people really like that idea of gain sharing for example, that they would have put that on the table and say, ‘Can we try this maybe for some sort of Orthopedic wait time procedures.’ or something like that, that’s where I would have started. I think just the negotiations have been so antagonistic the last couple rounds that there was very little room I think, to experiment with any sorts of major transformations like that, and policy like that right?” [PPM2]
Access to knowledge and information	“Also it was good reference because we were trying to implement funding policy. So, it wasn’t only about what it is, the alternative funding policy, but really how can we help the system going through the change and what [is the additional labour] that we need.. in order to make this things change.” [PPM4]
Process	
Planning	“I was always taking out some information… to evaluate on what was missing or plan the future steps and trying to get everyone on board.” [PPM4]
Opinion leaders	“…but the trouble with the policy dialogues is that they involve people who are already essentially committed to reform of kind or another. They may differ on the details. But they don’t get at the people who provide the money and the opportunities. So, I think the biggest barrier that I see now to real change is at the most senior level in government. And the reason is that A, they don’t actually have the time to understand the level at which it needs to be understood at. I mean, they just don’t have time. I feel really bad for the ministers. They’re just constantly putting out fires.” [PPM3]
Internal implementation leaders	“I think [individuals name], many of you will know this, did a fantastic job when she was pushing down generic drug prices the last time. Basically she said, “Why are we paying so much? Here’s the evidence.” She was able, as [position], to really, very effectively move forward. She personally bore a really heavy risk, or heavy burden in doing that, but I think it was actually in this case it was very effective by appointing an executive director, a political appointment, who ended up taking the heat instead of a politician, was quite an effective strategy for moving forward.” [R5]
External change agents	“… they’ve been able to use those information and put in place an expert panel - a three years expert panel - on the topic of activity based funding. To try to put-- to move and to bring this alive.” [PPM4]
Reflecting and evaluating	“There’s the old saw that you can’t manage what you can’t measure. We’re ready to start measuring a lot of things, not at the level of precision, perhaps not at the level of accuracy that we’d all like but it won’t start to get better until we do. And so I think the whole question of measurement is central to any type of reform that we consider. But more important I think it’s critical to think about what it is we want to measure. You know our health system is a reflection of who we are as a country so we should be measuring the things that are important to us, it’s our health system is one of the ways that we actually define ourselves positively.” [PPM1]

### Thematic analysis

#### Dialogue features

Informants felt the dialogues were well structured [see Table 2]. One informant felt that the *environment* provided an opportunity for “free-forum free-flow discussion … that, when you put people in rooms where they're wearing their hats and their titles you don't always have that kind of possibility” [NSO2]. Several informants described the environment as conducive to discussion and exploration of potential policy solutions [NSO2, PPM1, R03] on timely issues because a 10-year Health Accord between the provinces and federal government [33] was nearing completion and the possibility of a new accord was being discussed [FPM1]. Informants thought the dialogue participants represented “a *good cross-section of key players*” [FPM1] including individuals from “government, universities …various types of health organizations and non-governmental organizations” [R26]. The *expert knowledge syntheses* that were circulated as background to the dialogues and the presentations by their authors to set the stage for discussion [FPM1] were described as helpful because they provided in-depth analysis of each option in theory and practice.

#### Influence on capacity development

The opportunity to discuss “a whole range of issues” [NSO2] [see Table 3] enhanced *mutual understanding*. New policy solutions and *different ways of thinking* about the problem emerged from hearing the full range of perspectives and sharing participants’ experiences [NSO2]. Informants also described how they made important connections and developed *relationships* with others who they normally would not have had the chance to connect with. One informant commented that these types of events are designed to “make you feel good about yourself…that’s important because people … get a little bit more confident” [PPM3] to undertake action.

Over the medium term, informants described using what was learned at the dialogue within their organizations, for example, during “more sporadic briefing at the minister’s office” as a way to move the topic *forward in agenda setting* by saying “we’d had a chance to speak to a bunch of people across Canada…” [PPM2]. The discussions also helped in *policy formulation* because as one informant explained, they were “…in the back of my mind as we went through some … discussions at that time about how to do this [reform]” [PPM2]. The *face-to-face contact* made it easier to follow-up with people from other jurisdictions for clarification during policy development after the event [PPM3]. Several informants discussed how their organization was better *prepared when policy windows opened* and how “even if it's unpredictable - you have to have people who are ready and have the information, and have some commitment and understand how to do things.” [PPM3].

Few interview informants discussed longer-term capacity development at the systems level, which is not surprising since only 4 years had elapsed since the dialogues. However, researchers did comment on how the dialogues helped them better understand the priorities of policy-makers and to identify areas where new knowledge and new types of knowledge exchange products could significantly impact health systems over the longer term.

#### Influence on policy-making

Informants also described how these capacities influenced ideas, interests and institutions in policy-making [see Table 4].

Informants described how the dialogue discussions provided a chance to *view the broad policy landscape* by offering a “bird’s eye view” of different views around issues, expanded the range of policy ideas by “*seeding new ideas*” [FPM1] and helped “*socialize ideas*” by contextualizing research evidence to determine feasibility of options based on participant feedback [PPM1]. These all contributed to policy ideas that could shape how an issue was framed to advance it on the policy agenda.

Over the medium term, one federal policy-maker described how the dialogues *shaped options for discussion*, as (s)he was “factoring in” what (s)he had heard at the policy dialogue in terms of the “advice that I put up the line” [FPM1] during *policy formulation*. Informants also described how dialogue discussions helped *prioritize options and actions*, which helped policy-makers decide where to put their energies, based on “the relative importance of different mechanisms, or the relative likelihood of payoff” [PPM1]. One key informant stated “You could see when it was a policy option that was … embraced, that there [were] incentives to put them in place as opposed to other options where…they could see there were more limitations” [PPM4]. Informants described how a “*tightened consensus*” was developed among participants about what needs to happen [FPM1]. One informant described how the dialogue brought to light a “collective consciousness” [R7] among the policy-making and stakeholder communities that may have already been forming on these issues.

Similarly, the enhanced *mutual understanding* and *relationships* that were developed influenced *interests*. Informants described how the dialogue created “*a more cohesive policy community*” [R7] that was“*…*less confrontational, because more leaders from different sides of the equation knew each other” [NSO2] and that “communities [were] strengthened, just by opening more effective lines of communication” [NSO2] through *face-to-face connection*. Informants explained that non-attribution of comments made the dialogue an environment that was “non-confrontational; there are no bags of money on the table” [NSO2], where typically opposing groups could identify a common ground. A regional health authority decision-maker explained how attending the dialogue “… helped me build a little bit better bridges at the time, with the people … at our own ministry of health” [PPM3]. However, lack of *will for change* on the part of governments and stakeholder organizations was described as an impediment to advancing the policy issues being discussed at the dialogues.

Many informants felt that Canada’s *institutional* structure challenged major reform because “the more you have decentralization and devolution, the more, actually, the need for that central information requirement” [R11]. Several informants suggested that the federal government could play a greater role in *gathering and sharing evidence* and in *establishing benchmarks* and *common performance measures* at the national level to support system management and in evaluating any reforms undertaken [PPM1]. This could enable information about what was working in each jurisdiction to be shared and set the stage for pan-Canadian approaches, such as joint purchasing of pharmaceuticals.

Informants also discussed factors that influence policy implementation [Table 5]. One informant discussed how the *strength and quality of evidence* presented at a dialogue could either make a compelling case to take it forward or not [R08FG]. Another described how the dialogues helped informants understand the *relative advantage* of different financing options in terms of which has “the biggest potential payoff” [R01SI], the *adaptability* of options such as hospital funding approaches in rural settings, and the need for *flexibility* when it comes to physician payment depending on the context. Informants also discussed how the dialogues *packaged* evidence together as multiple options in a way that was more accessible to policy-makers. Finally, discussions of cost were seen to be important, particularly the *opportunity cost* of selecting one reform over another. In this way, the dialogues expanded participants’ knowledge and beliefs and self-efficacy to advocate for options within their organizations.

A number of themes related to the external context of the organizations represented at the dialogues were also discussed. For instance, several informants appreciated having a journalist present at one dialogue to represent public perceptions to convey *patient needs and resources*. The *cosmopolitanism* of participants’ organizations were enhanced through newly established relationships, which made it easier to follow up and ask questions about how implementation unfolded elsewhere. This also contributed *peer pressure* for reform in areas such as hospital funding. Many informants also pointed to a changing set of *external policy and incentives* that impeded implementation of many reform presented at the dialogues. Informants were clear that anticipation of a new funding agreement between the federal and provincial governments at the time of the dialogues was a key motivator for the dialogue discussions; however, momentum had been lost once the decision was made not to renew a health accord several months later [34]. This effectively closed a policy window because informants knew that there would “be lack of any kind of national … impetus behind these things” [PPM2], without which there is no strong incentive “… to provinces to have dialogue sessions … that can sort of bring them together” [PPM2]. As one informant stated, the dialogues were helpful at the time they were held, but given that the external environment for policy-making can change suddenly and make them less useful [PPM3].

Informants also discussed characteristics within organizations. Some were amenable to change through policy dialogues, while others were not. For example, the *rate of turnover of key staff* within governments and regional health authorities was pointed to as a structural factor that severely limited organizational capacity for implementing reform. In addition, the *culture* within government was described as being highly risk averse, with public servants facing a “web of rules” that could impede implementation [PSO2]. In contrast, the dialogues were seen as having the potential to shift the *relative priority* that organizations might give to different policy options and the extent of *leadership engagement*. For example, one key informant described how the spotlight being placed on hospital funding through the dialogue series encouraged one provincial finance minister to build on this momentum by hosting a similar dialogue at the provincial level and to establish an external advisory committee to advance reform. More often, however, informants felt that politicians and organizational leaders were not leading discussions about potential policy options. In part this reflected a lack of *tension for change* within healthcare organizations as the rate of growth of spending on healthcare began to diminish. Another challenge that could arise was having *available resources* to support implementation. On the other hand, the *knowledge and information shared* at the event were helpful in planning, *expanded knowledge and beliefs* about the proposed policy interventions and *self-efficacy* to undertake reform were enhanced.

From a process perspective, key informants described the potential for policy dialogues to assist with *planning* for implementation and about the importance of *engaging opinion* and *implementation leaders*. Lack of engagement of the most senior levels of government officials was described as “the biggest barrier to real change” [PB02SI] arising from most policy dialogues. For example, engaging an *external implementation leader* from outside government who “could take the heat” if anything went wrong was an effective strategy in one province. In another, the establishment of a 3-year expert panel as an *external change agent* following the dialogue facilitated implementation of hospital funding reform. The dialogues also led participants to *reflect on evaluation* approaches to meet the stated objectives of implementing reforms. There was considerable discussion about the need to be clear about the outcomes that were to be measured, including “measur[ing] the health gain” [R02] associated with proposed funding options in addition to economic impacts.

### Thematic synthesis, conceptual framework and pathways of influence

**Fig. 2:**
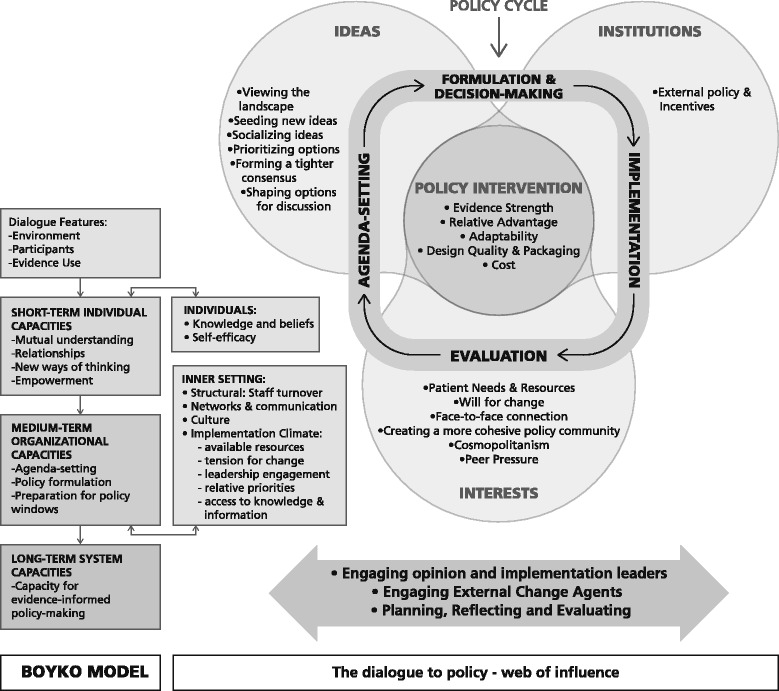
The dialogue to policy—web of influence. The dialogue to policy web of influence framework integrates elements of three frameworks from the published literature: the Boyko, 3-I framework and CFIR frameworks. The features needed for an effective policy dialogue and the capacities that can be developed through attending a policy dialogue (Boyko framework) are presented on the far *left*. Short-term individual and medium-term organizational capacities correspond to individual and organization inner setting factors identified in the CFIR framework shown immediately to the *right*. These capacities have the potential to influence the various stages of the policy cycle (agenda setting, formulation, implementation and evaluation) as they shape *ideas* of policy actors (policy-makers and other stakeholders) and their *interests* in light of *institutional* factors (the 3-I framework). The outcomes of policy processes pertain to the policy interventions at various stages of development across the cycle shown at the *centre* of the Venn diagram. The subthemes raised by study key informants are shown within each element of the conceptual framework and may link to other elements of the CFIR framework (e.g. external policy and incentives within *institutions* and patient needs and resources, cosmopolitanism and peer pressure within *interests*). The *double arrow* at the bottom underscores how engaging opinion and implementation leaders and external change agents during or in follow-up to a policy dialogue about the ideas discussed may support implementation processes of planning, reflecting and evaluation, consistent with CFIR. Similarly, the CFIR framework points to characteristics of policy interventions that influence their implementation and that can be part of the policy dialogue discussions

In Fig. 2, we present a conceptual framework that combines the thematic analysis across frameworks. It illustrates how dialogue features lead to capacities, which influence policy ideas, interests and institutions in agenda setting and policy formulation that can both shape and be shaped by contextual factors and processes that affect implementation.

**Fig. 3:**
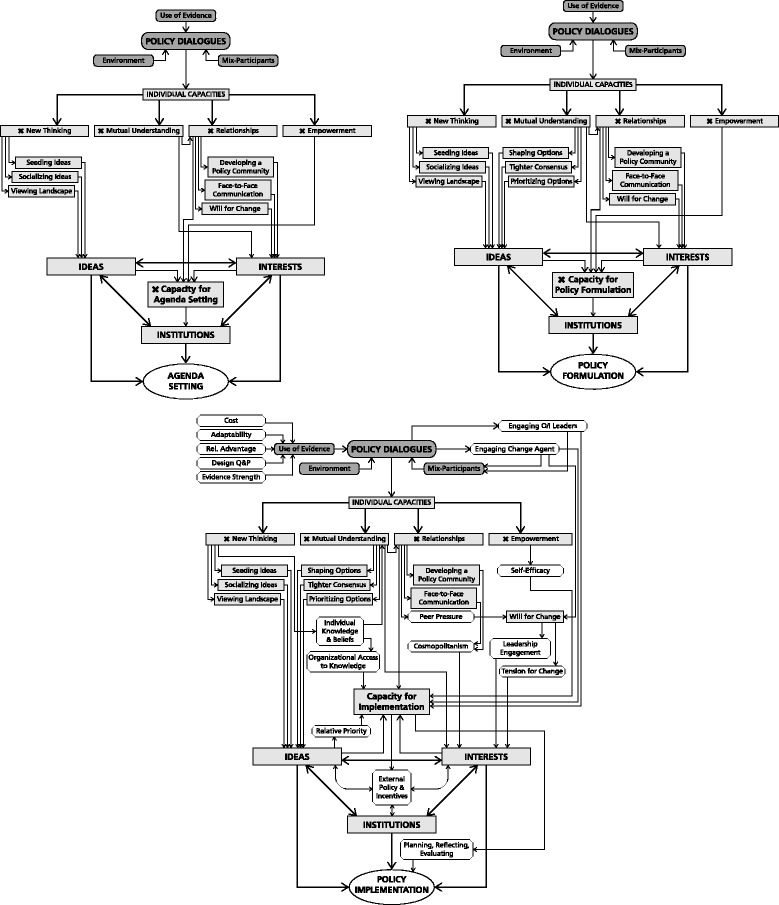
**a**–**c** Present potential pathways of influence of policy dialogues on policy-making by illustrating potential links between the various elements of the conceptual framework with policy-making at the agenda setting, formulation and implementation stages of the policy cycle respectively. Note that the suggested pathways do not imply direct causation but are meant to illustrate areas where policy dialogue designers may want to focus efforts to increase likelihood of influence of a given dialogue on policy processes. **a** Pathways of influence of policy dialogues on agenda setting. Illustrates potential pathways by which the capacities developed at policy dialogues can influence the ideas and interests within the 3-I framework and in turn the capacity to move different policy options onto public policy agendas at the agenda-setting stage of the policy cycle, as well as challenges to the influence of any given dialogue. The features of an effective policy dialogue are *shaded ovals* at the top of the diagram and the capacities developed from the Boyko framework are shown as *boxes* marked with an x. Potential linkages between capacities, the 3-I framework elements or the capacity for agenda setting are shown in *boxes with upper and lowercase text*. The elements of the 3-I framework (*uppercase font*) are illustrated as a triad of influence on the capacity for agenda setting. For example, with the right mix of features (evidence, participants and environment), policy dialogues can enhance new thinking by seeing and socializing ideas and providing a view of the policy landscape that influence *ideas* in circulation among policy-makers and stakeholders. Enhanced mutual understanding and relationships can foster a more cohesive policy community and face-to-face communication and strengthen the will to undertake reform among *interests.* These influences on ideas and interests and realities of the institutional setting, competing ideas and interests will together influence both capacity for agenda setting and which policy options make it onto policy agendas. **b** Pathways of influence of policy dialogues on policy formulation. Illustrates potential pathways by which the capacities developed at policy dialogues can influence *ideas* and *interests* within the 3-I framework and in turn the capacity to formulate policy solutions during the policy formulation stage of the policy cycle. The features of an effective policy dialogue are *shaded ovals* at the top of the diagram from the Boyko framework. The capacities described in the Boyko framework that are developed through policy dialogues are marked with an x**.** In addition to the capacities developed in Fig. 3a, a policy dialogue can enhance mutual understanding among dialogue participants that can help in shaping options, reaching a tighter consensus and assisting in setting priorities among options. These in turn can influence *ideas* and *interests*, which along with *institutions* can influence organizational capacity for policy formulation, and the policy options that are ultimately developed, as represented by the *oval* at the *bottom* of the figure. **c** Pathways of influence of policy dialogues on implementation. Illustrates potential pathways by which the capacities developed at policy dialogues can influence the implementation stage of the policy cycle. Constructs from the CFIR framework are represented by *unshaded rectangles with flattened corners*. For example, characteristics of interventions that influence implementation such as evidence strength, cost, adaptability, relative advantage and design and packaging of options can be shared as part of the evidence presented at the dialogue. Additionally, opinion leaders, implementation leaders and change agents can be engaged at or in follow-up to the dialogue to enhance likelihood of implementation. Building on the pathways of influence shown in Fig. 3a-b, new thinking enhances individual knowledge and beliefs about options that can contribute to organizational knowledge and priority setting, while relationships may lead to peer pressure and/or greater cosmopolitanism that along with enhanced self-efficacy can affect leadership engagement and tension for change within an organization. These factors affect organizational capacity for implementation including planning, reflecting and evaluating processes. External policy and incentives (including ideas, interests and institutions) and organizational capacity together will shape the likelihood and nature of policy implementation

In Fig. 3a–c, we illustrate and tease apart what one informant described as a “tangled web” [PPM1] of influence of policy dialogues on policy-making at the agenda setting, formulation and implementation stages of the policy cycle respectively.

Figure 3a illustrates pathways by which policy dialogues influence agenda setting. Through effective use of evidence, stakeholder representation and meeting environment, policy dialogues can encourage short-term individual capacities that include new ways of thinking, mutual understanding, relationships and empowerment. Potential pathways of influence on agenda setting are as follows:

- Pathway A1: Policy dialogues build participants’ individual capacities for new thinking by seeding and socializing new ideas and providing a view of the landscape that contributes to policy ideas that shape problem definition in policy-making and organizational capacity for agenda setting.
- Pathway A2: Policy dialogues build participants’ individual capacities for mutual understanding and relationships that can foster a more cohesive policy community and face-to-face connection and may create greater will for change among interests that contribute to organizational capacity for agenda setting.
- Pathway A3: Enhanced empowerment interacts with these influences on ideas and interests over time to promote greater organizational level capacity to advance policy problems onto the agenda.

Whether policy problems that make it onto the agenda move forward in the policy cycle will depend on other ideas, interests and institutional factors.

Figure 3b illustrates pathways through which policy dialogues can influence policy formulation. In addition to the influence of participants’ enhanced mutual understanding on policy ideas and interests discussed above with respect to agenda setting,

- Pathway B: Enhanced mutual understanding developed at policy dialogues can assist in shaping options for discussion, prioritizing options and creating a tighter consensus around ideas that can enhance organizational capacities over the medium-term not only for problem definition but also for formulation of policy solutions.

Figure 3c illustrates pathways through which policy dialogues can influence policy implementation. In addition to the influences of dialogues on agenda setting and policy formulation discussed above, there are a number of pathways by which policy dialogues may influence implementation by expanding (i) knowledge of key characteristics of options and shaping (ii) individual characteristics, (iii) organizational characteristics, (iv) the outer setting of organizations and (iv) implementation processes.

- Pathway C1: The evidence presented at policy dialogues can include information about the relative advantage, cost, adaptability and evidence strength of different policy options and package them in a way that may be more attractive for implementation.
- Pathway C2: Greater individual empowerment can lead to greater self-efficacy and enhanced organizational capacity for implementation.
- Pathway C3: Individual capacities for new thinking can enhance individual knowledge and beliefs about the options that enhance mutual understanding, contribute to a tighter consensus about policy ideas and shape organizational priorities, as well as organizational access to knowledge and information to support organizational capacity for implementation.
- Pathway C4: The relationships developed at the dialogue that create a more cohesive community can also foster greater cosmopolitanism and peer pressure that may contribute to tension for change, enhancing organizational interest in policy implementation.
- Pathway C5: The external policy context of organizations as shaped by the interacting forces of ideas, interests and institutions may influence the extent to which organizations are successful in advancing knowledge shared at policy dialogues at all stages of the policy cycle, including implementation.
- Pathway C6: Policy dialogues have the potential to influence implementation processes by engaging opinion and implementation leaders and external change agents and providing input to organizational processes of planning, reflecting and evaluating. Leadership engagement can also help to establish greater tension for change within organizations and engage organizational leaders as interests in policy implementation.

Informants also pointed to several organizational contextual factors that are less amenable to influence through policy dialogues such as staff turnover, poor communication and a risk adverse organizational climate.

#### One element in an incremental process

While informants described policy dialogues as “an important part of the process of policy change”, along with “generating useful research … hearing views and doing appropriate policy analysis” [FPM1], they also indicated that it is difficult to attribute any direct causal relationship, because policy moves often moves slowly over time.

It's hard to really point to something really specific that this particular series of conversations gave rise to. These areas of policy, they change so slowly over time, and it's really kind of a building thing, an accumulation of pieces of work, of conversations, of positioning by stakeholders and governments, and you see movement over time. [FB11SI]

Dialogues were described as contributing to a continuing conversation that may last many years within the public debate before any action is taken. One informant described the work as being “extremely influential” and “picked up by a lot of other people,” but that “…it's just overall, a whole lot of different pieces of work that come together to give you a picture” [R4].

This involved a whole bunch of motivated people. We're taking some good pieces of research. We're talking about how those things influence decision-making, but at the end of the day, no one in government is going to stand up and say, It is because of - only because of - the discussion that was at this conference. [PPM1]

This temporal aspect is difficult to capture in the figures because the journey through the policy cycle is non-linear and may be disrupted as priorities shift, for example, due to external events, shifting political considerations, realignment among interests [17] or poor evaluation results that send a policy back to an early stage of the policy cycle.

## Discussion

Our findings support the role that policy dialogues can play in developing individual, organizational and system level capacities for policy reform, as has been discussed in the literature [8, 9, 35, 36], and illustrate the multiple factors that can influence whether they impact policy decision-making, typically through an incremental influence on policy processes over time. The analysis suggests that the intersection of interests, institutions and ideas, as well as characteristics of individuals, organizations, policy options and the external environment and implementation processes can influence the impact of a policy dialogue on capacity development and policy-making. It is also important to recognize that many of the factors described in Fig. 3a–c that can be influenced by policy dialogues can also act as barriers to influencing policy decision-making at different stages of the policy cycle. For example, some options discussed at dialogues do not move forward because of weak evidence, lack of will for reform, insufficient leadership engagement, or lack of self-efficacy or peer pressure.

By teasing apart these mechanisms, the findings point to considerations that designers of policy dialogues may want to target in order to meet specific objectives related to the stage of the policy cycle. It may be beneficial for organizers to reach out to participants prior to the dialogue to assess the contextual factors they face in advancing options at the individual, organizational and external policy levels and tailor discussions at the events accordingly. This could include asking participants to reflect on their own readiness for implementation, the implementation climate of their organizations and external environment and potential implementation processes. Such advance preparation may enable the discussions at the dialogue to focus on what it takes to move evidence into practice to inform various stages of policy development across the policy cycle. If the objective is to help to move an issue onto the policy agenda or shape ideas for policy formulation, the range of evidence and expert input should ensure that participants have the opportunity to view the full landscape of stakeholder perspectives, to hear new ideas, and that the structure of the discussions create processes by which evidence can be “socialized” and prioritized into feasible policy options [17]. If the objective is to influence implementation, the selection of dialogue participants should include senior decision-makers, opinion leaders and external change agents, or they should be targeted in follow-up activities to contribute to the tension for change that can shift organizational priorities [4]. The findings also suggest that there needs to be a realistic consideration of the contextual influences before embarking on policy dialogues intended to promote reform, to avoid wasting resources if there is an absence of organizational capacity and system level will for change or if institutional factors such as external policies and incentives or performance measures are not in place. The framework may be especially helpful in future work to more clearly evaluate the success or failure of dialogues against stated goals in light of the policy and organizational context.

The model makes an important contribution by helping to address gaps in the implementation literature identified by Bowden and colleagues [10] with respect to policy considerations, such as the influence of evidence on policy networks, the need to consider capacity for implementation at multiple levels, while recognizing that effective knowledge transfer is not a “one off” event, but instead a powerful and continuous process in which knowledge accumulates and influences thinking over time.

At the same time, this research is subject to several limitations. In any study of this kind, there are concerns about how well interview informants will recall the content discussed at dialogues held several years earlier and how this affects their interpretation of subsequent policy developments. In addition, there may be other contextual influences beyond those mentioned here.^2^ There is also the risk that study participants feel more positive about the influence of policy dialogues than those who declined to participate. Finally, the findings rest on a single case involving four national level dialogues in Canada related to healthcare financial sustainability. The framework may serve as a basis for future research to test the proposed pathways of influence developed here while studying the influence of policy dialogues in other contexts.

## Conclusions

The findings provide a conceptual framework and illustrate pathways by which policy dialogues can advance the use of evidence to inform policy content and processes. Particular attention should be placed on the intended objective of the dialogue in influencing different stages of the policy cycle, while creating a dialogue environment that can help participants to expand capacities. Strategic thought must be given to the characteristics of the organizational and political context to assess whether there is potential for knowledge gained at the dialogue to be put into practice.

## Abbreviations

CFHI: Canadian Foundation for Healthcare Improvement
CFIR: Consolidated Framework for Implementation Research
HFiT: Healthcare Financing, Innovation and Transformation
iHEA: International Association of Health Economics Conference

## Informant codes

FPM: Federal policy-maker
PPM: Provincial policy-maker
NSO: National stakeholder organization
PSO: Provincial stakeholder organization
R: Researcher

## Footnotes

1 The views are reported in [37]

2 For example, there are elements of the CFIR model that were not mentioned by our informants, such as intervention complexity, organizational incentives and rewards, goals and feedback, learning climate and available resources individual stage of change, extent of identification with the organization and other personal attributes. It is unlikely these factors would be shaped by capacities developed at a dialogue and so are not expected to be a serious omission from the framework.
